# Guaranteeing girls’ right to education: an imperative for global brain health

**DOI:** 10.1590/1980-5764-DN-2025-0319

**Published:** 2025-08-04

**Authors:** Timothy Daly

**Affiliations:** 1 University of Bordeaux, Bordeaux Population Health, Bordeaux, France.; 2 University of Bordeaux, ImmunoConcept, Bordeaux, France.; 3 Bioethics Program, FLACSO Argentina, Buenos Aires, Argentina.

Dear Editor,

The March 2025 Report by the United Nations Children’s Fund (UNICEF), *Girl Goals: What has changed for girls? Adolescent girls’ rights over 30 years* ^*1*^ , on education, violence, and health, has wide-reaching implications that include population brain health.

The report found that, despite important progress, almost four out of ten adolescent girls worldwide do not finish upper secondary school, particularly those from poor, rural, and marginalised populations^1^. As well as representing major missed life opportunities for millions of children worldwide, these low numbers also matter for brain health: the meta-analysis of the 2024 Lancet Commission on dementia found that low educational attainment is consistently the most robust modifiable risk factor for dementia, a syndrome of pathological age-related cognitive decline that is a leading cause of morbidity worldwide^2^. Moreover, women with dementia outnumber men two-to-one, and regions where cases are expected to increase the most by 2050-the Middle East and sub-Saharan Africa^3^ - correspond to those in which girls’ right to education is the least respected.

In our contemporary period, health inequalities have reached a crisis point^4^. In particular, the idea of the right to health is being eroded worldwide. For instance, the United States has recently withdrawn from the World Health Organization (WHO)^5^, whose core mission has always been to promote the right to health for everyone^6^. To resist short-sighted political impacts on brain health policy making, a human rights-based approach to brain health promotion^7^ ^,^ ^8^ that adopts a life-course understanding of dementia^2^ is needed. Importantly, education quality as well as quantity is important to maximise the benefits of education on brain health^9^.

Respecting, protecting and fulfilling the human right to education for girls and boys worldwide will take us collectively closer to equitable brain health promotion^10^. Guaranteeing such access to education is a global imperative for both girls’ rights and women’s brain health and has never been more urgent in our divided world with its aging population.
